# Optogenetic mutagenesis in *Caenorhabditis elegans*

**DOI:** 10.1038/ncomms9868

**Published:** 2015-12-03

**Authors:** Kentaro Noma, Yishi Jin

**Affiliations:** 1 Howard Hughes Medical Institute, University of California, San Diego, La Jolla, California 92093, USA; 2 Section of Neurobiology, Division of Biological Sciences, University of California, San Diego, La Jolla, California 92093, USA; 3 Department of Cellular and Molecular Medicine, University of California, San Diego, La Jolla, California 92093, USA

## Abstract

Reactive oxygen species (ROS) can modify and damage DNA. Here we report an optogenetic mutagenesis approach that is free of toxic chemicals and easy to perform by taking advantage of a genetically encoded ROS generator. This method relies on the potency of ROS generation by His-mSOG, the mini singlet oxygen generator, miniSOG, fused to a histone. *Caenorhabditis elegans* expressing His-mSOG in the germline behave and reproduce normally, without photoinduction. Following exposure to blue light, the His-mSOG animals produce progeny with a wide range of heritable phenotypes. We show that optogenetic mutagenesis by His-mSOG induces a broad spectrum of mutations including single-nucleotide variants (SNVs), chromosomal deletions, as well as integration of extrachromosomal transgenes, which complements those derived from traditional chemical or radiation mutagenesis. The optogenetic mutagenesis expands the toolbox for forward genetic screening and also provides direct evidence that nuclear ROS can induce heritable and specific genetic mutations.

## Abstract (web summary)

Inducing random mutation of *C. elegans* DNA is a widely used technique to investigate gene and protein function. Here the authors introduce a method of optogenetic mutagenesis, driving the generation of reactive oxygen species, which avoids the use of toxic chemicals.

## Graphical abstract

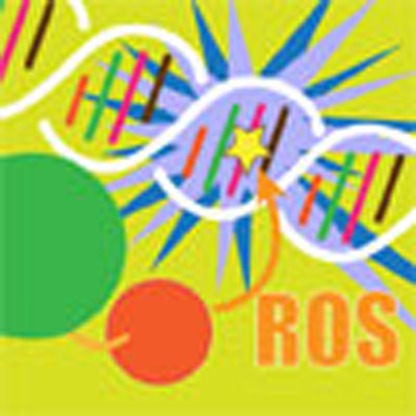

Reactive oxygen species (ROS) are known to damage DNAs and proteins, induce oxidative stress, and trigger a plethora of protective responses in cells or organisms^1^. In recent years, a number of genetically encoded photosensitizers, such as KillerRed, SuperNova, and mini singlet oxygen generator (miniSOG) have been developed to manipulate ROS *in vivo* for a variety of purposes^2^. Among these, miniSOG, a 106 amino-acid monomeric protein, was originally reported to produce singlet oxygen upon blue light exposure^3^, and may also produce ROS other than singlet oxygen^4^,^5^. MiniSOG has been used for correlative light and electron microscopy to visualize tagged proteins within tens of nanometers of their subcellular residence^3^. The ability of miniSOG to locally generate ROS has also been exploited to ablate cells^6^ and to inactivate proteins^7^ in a blue light dependent manner. Such applications have facilitated investigations of neural circuits and synaptic transmission.

ROS are also known to modify DNA^1^. For example, chemically induced ROS was shown to cause a range of mutations in bacterial phage DNA^8^,^9^. However, to our knowledge, it is not yet known whether genetically encoded photosensitizers can produce heritable DNA mutations in multicellular organisms. Here, we demonstrate that miniSOG fused to a histone (His-mSOG) induces heritable mutations in a light-dependent manner in *C. elegans*. We refer to the mutagenesis using photoactivation of genetically encoded proteins as ‘optogenetic mutagenesis'. This method is rapid, simple and free of toxic chemicals. Optogenetic mutagenesis produces a wide variety of changes in DNA such as single-nucleotide variants (SNVs), deletions, and insertion of extrachromosomal transgenes. The distinctive mutagenic spectrum of His-miniSOG makes it a valuable complement to traditional chemical or radiation mutagenesis. Additionally, our observations demonstrate that high levels of ROS in the nucleus can cause specific and genetically heritable mutations.

## Results

### His-mSOG mutagenizes the genomic DNA

**Figure 1:**
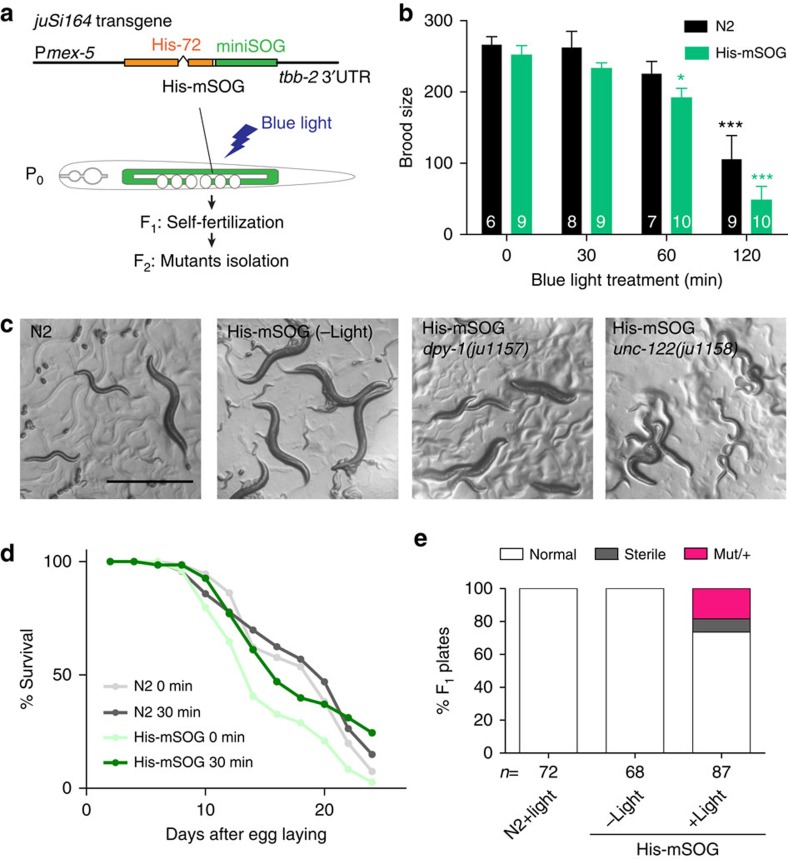
His-mSOG induces heritable mutations in a light-dependent manner. (**a**) Schematic of the HIS-72::miniSOG construct and optogenetic mutagenesis procedure. Parental (P_0_) His-mSOG worms expressing a single copy transgene *juSi164* in the germline are treated with blue light. Mutants are isolated from the F_2_ generation (Mut/Mut) after self-fertilizing F_1_ (Mut/+). (**b**) The brood size of wild-type and His-mSOG animals treated with blue light for different durations at gravid young adult stage. The brood sizes of N2 and His-mSOG worms were comparable and were decreased by prolonged blue light treatment. Error bars indicate S.E.M. Statistics: One-way ANOVA with Bonferroni post-test for comparison of light duration, *P<0.05, ***P<0.001. Two-tailed *t*-test for comparison between N2 and His-mSOG (*P*>0.05). (**c**) Bright-field images of animals with genotype and condition indicated. Untreated His-mSOG worms are indistinguishable from the wild type. Two mutants with visible phenotypes, Dumpy and Uncoordinated, isolated after light treatment of His-mSOG strains. Scale bar: 500 μm. (**d**) Survival curve of wild-type and His-mSOG worms with and without blue light treatment at young adult stage. *n*=4 experiments. (**e**) Frequency of miniSOG-induced mutants in F_1_ progeny. Young adult parental worms were treated with or without blue light for 30 min and F_1_ animals were isolated. ‘Mut/+' and ‘sterile' indicate the fraction of F_1_ animals segregating progeny with visible phenotypes and no progeny, respectively. *n*=number of F_1_ animals.

We reasoned that activation of miniSOG in proximity to DNA could generate mutagenic ROS. To test this idea we fused miniSOG to the C-terminus of a histone H3.3 variant, *C. elegans* HIS-72 (ref. 10) (His-mSOG). To express His-mSOG in the germline we generated a single-copy integrated transgene *juSi164[Pmex-5-HIS-72::miniSOG-3'UTR(tbb-2)]* using the *mex-5* promoter and the 3' untranslated region of *tbb-2* (ref. 11) (Fig. 1a). Without blue light treatment, His-mSOG animals had normal growth rate and brood size (Fig. 1b, His-mSOG, 0 min light), displayed wild-type body shape and movement (Fig. 1c), and lived a normal lifespan (Fig. 1d, His-mSOG, 0 min light). We activated miniSOG by illuminating young adult hermaphrodites with blue light and examined if they produced mutant progeny. The blue light condition used was 2.0 mW/mm^2^ for 30 min with a 460-nm wavelength (see Methods), which did not affect the brood size or lifespan of N2 wild-type or His-mSOG animals (Fig. 1b,d, 30 min light). Among the F_2_ progeny from the light-treated His-mSOG parents, we found mutant animals with visible phenotypes such as uncoordinated and dumpy (Fig. 1c). We singled 87 F_1_ hermaphrodites, and observed that 10% of them were sterile and 20% gave F_2_ progeny with visible mutant phenotypes in the light-treated His-mSOG group (Fig. 1e). Most of these phenotypes were inherited in the following generations, suggesting that they were caused by genetic mutations in the germline or early embryos. Wild-type animals treated with blue light did not produce visible mutants, nor did His-mSOG animals without exposure to blue light (Fig. 1e), indicating that the observed mutagenesis is dependent on the combination of blue-light exposure and the His-mSOG transgene.

We used outcrossing and genetic mapping to determine whether the observed phenotypes were due to mutations in single genes. A dumpy mutation *ju1157* was mapped to the left arm of chromosome III in the course of outcrossing, and failed to complement *dpy-1(e1)*^12^. We determined by Sanger sequencing that it contained a 442-bp deletion in *dpy-1*. An uncoordinated mutation *ju1158* was mapped to the right arm of chromosome I. By whole-genome sequencing (WGS), we found a dinucleotide GA was mutated to C in the *unc-122* gene^13^, resulting in a frameshift. *ju1158* failed to complement *unc-122*(*e2520).* Thus, optogenetically induced mutations can attribute to single genes.

### Frequency of optogenetically induced mutants

**Figure 2:**
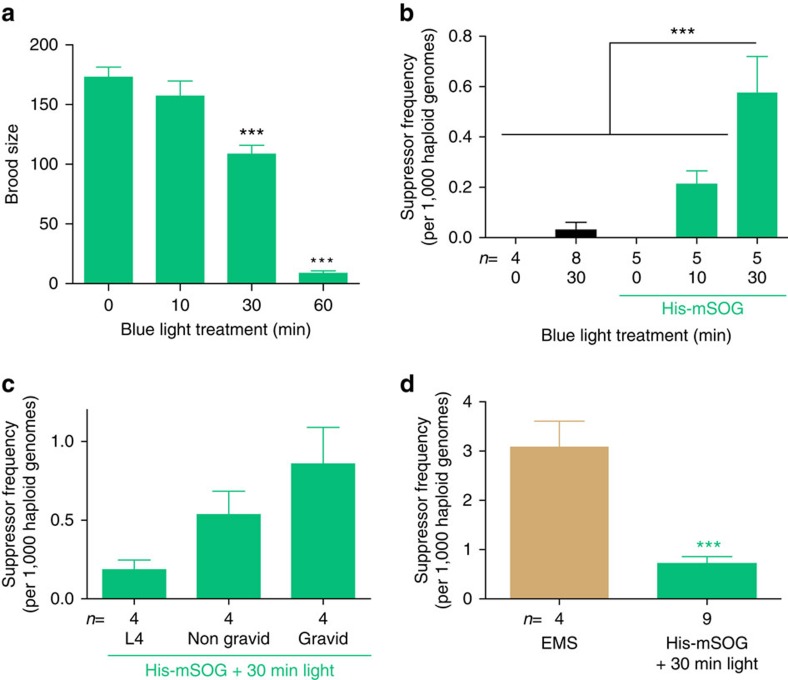
Efficiency of His-mSOG mutagenesis based on the *rpm-1(lf); syd-2(lf)* suppressor screen. The mutation rate of His-mSOG mutagenesis was estimated by the number of suppressor mutations isolated from screening in the *rpm-1(lf); syd-2(lf)* background as described in Methods. (**a**) Brood size of CZ20638 *juSi164 unc-119(ed3); juIs1; rpm-1(lf); syd-2(lf)* after exposure to different duration of blue light at gravid young adult stage. n=10 animals. (**b**) Effect of light dosage. The frequency of mutations of His-mSOG-mutagenesis at gravid young adult stage was determined for different durations of light exposure. Negative controls are non-transgenic *rpm-1(lf); syd-2(lf)* animals with and without light exposure. (**c**) Effect of developmental stage. His-mSOG-carrying animals were treated with blue light for 30 min at different developmental stages. (**d**) Comparison between the efficiency of EMS and His-mSOG. The mutation rate of EMS mutagenesis was determined from 50 mM EMS treatment of CZ1338 *juIs1; rpm-1(lf); syd-2(lf)* and compared to His-SOG-mutagenesis with 30-min light exposure at gravid young adult stage. Error bar indicates S.E.M. (**b**–**d**) n=number of independent experiments, 20 P_0_ per experiment. Statistics: (**a**,**b**) One-way ANOVA, ***P<0.001. (**d**) Two-tailed *t*-test, ***P<0.001.

We next investigated the frequency of optogenetically induced mutations using a genetic suppressor screen for the locomotion defects of *rpm-1(lf); syd-2(lf)* double mutants^14^. This screen has three advantages: first, well-moving suppressor mutants are easily identified among the severely uncoordinated *rpm-1(lf); syd-2(lf)* animals; second, saturation EMS screens have identified only a small number of suppressor loci that are distributed in three chromosomes^14^; third, it allows us to quantitatively compare the efficiency and spectrum of optogenetically induced mutations with those of previously characterized EMS-induced mutations^14^. The frequency of mutations was calculated from the number of independent suppressors and of the mutagenized haploid genomes based on the average brood size (see Methods). In the *rpm-1(lf); syd-2(lf)* background, the brood size of His-mSOG strain was dramatically decreased when animals were exposed to blue light for >30 min (Fig. 2a). Therefore, we illuminated these animals with blue light for 0, 10 and 30 min, and found that the frequency of mutations increased at higher light dosage (Fig. 2b). The *rpm-1(lf); syd-2(lf)* strain lacking His-mSOG transgene displayed a much lower mutation rate even with 30-min blue light exposure (0.03±0.03/1,000 haploid genomes, *n*=8 independent experiments, total ∼64,000 mutagenized haploid genomes, Fig. 2b). When *rpm-1(lf); syd-2(lf)* animals with His-mSOG were maintained continuously under ambient light, we found only one suppressor mutation among the progeny of ∼70,000 F_1_ animals, which is comparable to the spontaneous mutation rate in *C. elegans*^15^. These data support a conclusion that optogenetic mutagenesis is specific to the interaction of His-mSOG and blue light.

In *C. elegans* it is highly recommended to mutagenize animals at fourth larval (L4) stage when germline proliferation is at its peak^16^. We examined the efficiency of optogenetic mutagenesis at different developmental stages, and found that gravid young adults showed a higher mutation frequency than L4 under the 30-min blue light illumination protocol (Fig. 2c). Compared to chemical mutagens, which may not efficiently pass through adult cuticles and/or egg shells, blue light might efficiently affect the transparent eggs in optogenetic mutagenesis.

Based on nine independent suppressor screens of *rpm-1(lf); syd-2(lf)* using the 30-min illumination protocol, we determined the mutation frequency of His-mSOG mutagenesis to be 0.72±0.14 per 1,000 haploid genomes (Fig. 2d, total ∼78,000 mutagenized haploid genomes). This mutation rate did not change after maintaining the His-mSOG transgenic strain over ten generations, suggesting that the germline expression of His-mSOG is not silenced over time or generations. We further compared the mutagenesis efficiency of His-mSOG to that of EMS, the most commonly used chemical mutagen in *C. elegans*^12^. We treated *rpm-1(lf); syd-2(lf)* animals without His-mSOG transgenes (CZ1338) with a standard 50 mM EMS protocol, which is known to give rise to one loss-of-function mutation of any particular gene out of 2,000 mutagenized haploid genomes^12^ (see Methods). We calculated the mutation rate of EMS in our screen to be 3.1±0.52 (*n*=4 independent experiments, total ∼21,000 mutagenized haploid genomes), about 4.4 times higher than that of His-mSOG optogenetic mutagenesis using 30-min illumination protocol (Fig. 2d). It might be possible to increase the efficiency of optogenetic mutagenesis by increasing the duration of light exposure in a wild-type background since wild-type animals are more tolerant to blue light exposure than are *rpm-1(lf); syd-2(lf)* animals (Figs 1b and 2a).

### Frequency of optogenetically induced SNVs

**Figure 3:**
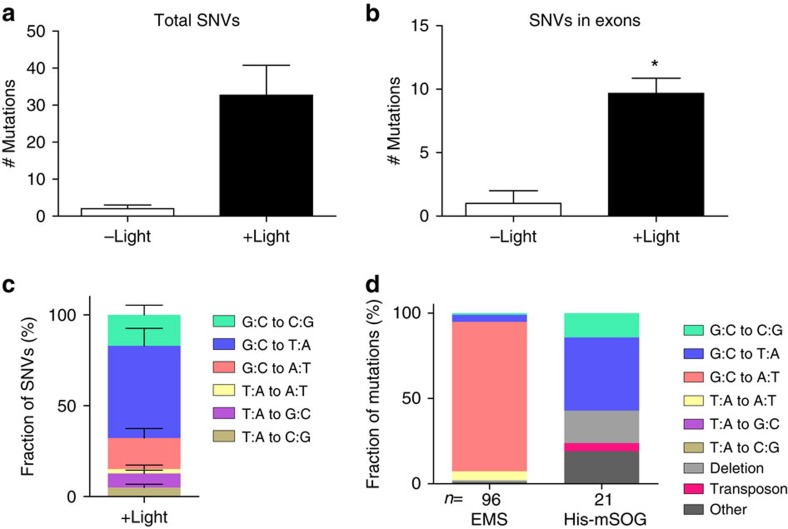
His-mSOG mutagenesis induces SNVs and deletions. (**a**–**c**) Two untreated non-suppressors and three light-induced suppressors from the *rpm-1(lf); syd-2(lf)* suppressor screen were analyzed by WGS. (**a**) Total unique homozygous SNVs. (**b**) Unique homozygous SNVs in exons. Statistics: Two-tailed *t*-test, (**a**) *P*=0.061, (**b**) *P<0.05. (**c**) Types of SNVs in the three light-induced suppressors. (**d**) Comparison of types of mutations generated in His-mSOG- and EMS- mutagenesis. The causative mutations of *rpm-1(lf); syd-2(lf)* suppressors were categorized. ‘Other' includes possible large deletions and/or chromosomal rearrangements. *n*=number of suppressor mutants.

The mutation frequency described above is estimated from the specific suppressor loci. To determine the genome-wide frequency of optogenetically induced mutations, we carried out WGS analysis. We obtained >20-fold genome coverage for three groups of strains: (1) the parental His-mSOG strain (CZ20638 *juSi164; juIs1; rpm-1(lf); syd-2(lf))*; (2) three optogenetically-induced suppressor strains from the parental strain; (3) two isogenic siblings from CZ20638 that were not exposed to the blue light but were handled in parallel to the three suppressor strains. We determined the unique homozygous SNVs using Galaxy platform (see Methods)^17^. The non-mutagenized strains contained 2.0±1.0 total SNVs (Fig. 3a), and none of them were in exons (Fig. 3b), compared to the parental strain. This is within the range of the previously reported rate of spontaneous mutations in wild-type *C. elegans*^18^. In conjunction with the phenotype-based mutation rate (Fig. 2b), we conclude that His-mSOG does not induce mutations without blue light exposure. In contrast, the three optogenetically induced suppressor strains had increased numbers of total SNVs and SNVs in exons (37.0±8.1 and 9.7±1.2, respectively, Fig. 3a,b). By comparison with previous studies^19^,^20^, we estimate that His-mSOG mutagenesis induces approximately ten times fewer SNVs than 50 mM EMS mutagenesis. 26% of *C. elegans* genome is known to contain exon sequences^21^. Although histone might have different distribution between exons and non-exons, we did not see any bias toward exon regions (SNVs in exons/total SNVs=9.7/37=0.26). We found about eight non-synonymous SNVs in each optogenetically induced suppressor strain. Table 1 lists these nucleotide changes and predicted amino-acid changes of genes affected. Among non-synonymous SNVs, we found mutations of *dlk-1* or *pmk-3* in two suppressor strains. Complementation tests confirmed that these mutations caused loss of function in each gene. These analyses show that His-mSOG can modify functions of genes at a suitable frequency for mutagenesis.

### Spectrum of optogenetically induced mutations

To determine the types of mutations of His-mSOG mutagenesis, we first examined the genome-wide landscape of optogenetically induced SNVs by analyzing WGS data for three *rpm-1(lf); syd-2(lf)* suppressors described above. We found that these SNVs showed a high ratio of G:C to T:A transversions (45.7±5.6%, *n*=3 suppressors, Fig. 3c), while G:C to C:G transversions and G:C to A:T transitions were also detected (17.4±3.0% and 17.4±3.0%, respectively, *n*=3 suppressors, Fig. 3c). We further characterized causative mutations of eighteen additional *rpm-1(lf); syd-2(lf)* suppressors by complementation tests and Sanger sequencing (Table 2 and see Methods). Optogenetically induced SNVs tended to be G:C to T:A and G:C to C:G transversions, accounting for 42.9% and 14.3% of causative suppressor mutations, respectively (Fig. 3d).

We next examined deletion mutations. Through analysis of WGS data, we found several uncovered regions of >200-bp in the optogenetically induced suppressors. We confirmed two of these uncovered regions to be a 437-bp deletion and a 1,427-bp deletion plus 787-bp insertion by Sanger sequencing (Supplementary Fig. 1). Furthermore, we found by Sanger sequencing that four optogenetically induced alleles of *rpm-1(lf); syd-2(lf)* suppressors were deletions of 1, 16, 98, and 320 bp, accounting for 19% of the causative mutations (Fig. 3d Deletion, Table 2). These results suggest that His-mSOG can induce deletions of a few to several hundred base-pairs. The rate of deletions in His-mSOG mutagenesis is higher than EMS mutagenesis, in which we obtained only one 26-bp deletion allele among 96 suppressor mutations, corresponding to 1% of the causative mutations^14^ (Fig. 3d). Four optogenetically mutated strains including one whole-genome-sequenced strain (CZ22337, *ju1291*, Table 1) appeared to contain other types of mutations such as larger deletions and/or chromosomal rearrangements (Fig. 3d Other and Table 2). One of the optogenetically induced *dlk-1* alleles (*ju1203*) contained a Tc3 transposon insertion in the exon 4, which disrupts the kinase domain of DLK-1 protein (Fig. 3d and Table 2) although transposons are thought to be inactive in the germline in the N2 wild-type strain^22^. Overall, our data demonstrate that His-mSOG mediated mutagenesis causes a broad spectrum of mutations.

### Distribution of optogenetically induced mutations

**Figure 4:**
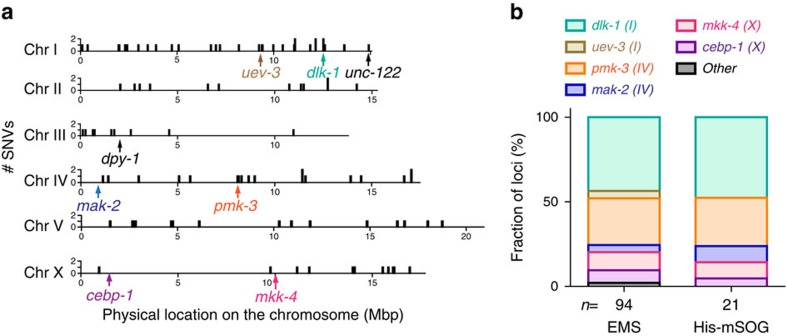
His-mSOG mutagenesis is unbiased with respect to chromosomes or loci. (**a**) Physical locations of optogenetically induced SNVs on chromosomes. Total SNVs were obtained from the WGS analysis of three light-induced *rpm-1(lf); syd-2(lf)* suppressors. The reads were pooled, divided into 10,000-bp bins and plotted at the location on the physical map of each chromosome. The physical location of six major *rpm-1(lf); syd-2(lf)* suppressors and two characterized optogenetically induced mutants, *dpy-1* and *unc-122*, are indicated as colored arrows. (**b**) The fraction of loci from EMS^14^- and His-mSOG-mutagenesis in the *rpm-1(lf); syd-2(lf)* suppressor screen. *n*=number of suppressor mutants.

We asked if His-mSOG-mutagenesis displayed any chromosomal bias since X chromosome is known to become depleted in Histone 3.3 including HIS-72 during gametogenesis^10^. Figure 4a shows the chromosomal distribution of optogenetically induced SNVs based on WGS analysis of three *rpm-1(lf); syd-2(lf)* suppressors described above. There were more SNVs on chromosomes I and IV because of the linkage to the causative suppressor mutations in *dlk-1* (LG I) and *pmk-3* (LG IV), respectively. Nonetheless, optogenetically induced SNVs showed relatively equal distribution on all chromosomes including X, suggesting that His-mSOG mutagenesis is not strongly biased with respect to chromosomes or positions on the chromosomes (Fig. 4a). The lack of bias was also observed in the analysis of the optogenetically induced loci of the *rpm-1(lf); syd-2(lf)* suppressors. Our optogenetic mutagenesis hit five out of six major known suppressors. Moreover, the relative frequency of the suppressors obtained from His-mSOG-mutagenesis matched that observed in our previous saturation EMS screens (Fig. 4b)^14^. Thus, we conclude that His-mSOG mutagenizes all the chromosomes without obvious positional bias.

### Optogenetic integration of multi-copy transgenes

A common practice in the making of transgenic reporter lines is to integrate extrachromosomal high-copy transgenes into the genome to achieve stable and consistent expression. Methods such as UV irradiation with trimethylpsoralen (TMP) and gamma irradiation^23^ are widely used for transgene integration but require the use of toxic chemicals and/or special equipment. These methods are thought to induce chromosome breaks, which lead to transgene integration. His-mSOG-mutagenesis also appears to create chromosome breaks because it produced deletions (Fig. 3d, see Discussion), suggesting that His-mSOG mutagenesis could be applied for integration of transgenes. To test this idea, we made transgenic worms expressing the *rol-6(su1006)* genomic fragment, which causes a dominant roller phenotype^24^, in the background of His-mSOG transgene. After blue light illumination for 30 min, we obtained an average of one integrated line from 200 F_1_ animals in three independent experiments (Supplementary Fig. 2a). These transgenes were confirmed to be chromosomal integrations based on Mendelian segregation after outcrossing (∼75% roller animals in the F_2_ generations, Supplementary Fig. 2b). The efficiency of His-mSOG-based integration is comparable to the traditional UV/TMP integration in our hands, suggesting that optogenetic mutagenesis is useful for chromosomal integration of transgenes.

## Discussion

We have established an optogenetic approach for genome-wide mutagenesis that avoids the use of toxic chemicals such as EMS, ENU or TMP. Unlike chemical or radiation mutagenesis, only dozens of parental (P_0_) animals are needed for blue light illumination using a simple and inexpensive LED setup, and the mutagenesis process is complete within an hour. Since the mutagenic effect of His-mSOG is dependent on strong light, His-mSOG strains without blue light exposure are more stably maintained than mutator strains, which induce high frequency of spontaneous mutations by transposon activation in the germline^25^. Furthermore, His-mSOG mutagenesis can complement other methods because it induces a different spectrum of mutations, compared to those induced by traditional chemical or radiation mutagenesis^20^. For example, EMS-induced mutations are strongly biased toward G:C to A:T transitions^15^,^20^, comprising 87.5 % of the mutations induced in our screen^14^ (Fig. 3d). Therefore, some codons are rarely induced (CCX for Pro and GGX for Gly) or mutated (TTW for Phe, ATYand ATA for Ile, TAY for Tyr, AAY for Asp, and AAR for Lys) in EMS mutagenesis. In contrast, we found these substitution mutations in His-mSOG mutagenesis: Lys150Asn and Ala451Pro in PMK-3, and Tyr215Stop in CEBP-1 (Table 2). We further note that all the optogenetically induced SNVs in the *rpm-1(lf); syd-2(lf)* suppressor screen affect amino acid residues different from those affected in the suppressor mutations previously found after EMS mutagenesis^14^. These substitution mutations provide insights into protein functions. For instance, the PMK-3(Lys150Asn) mutation affects the conserved ATP-binding site in the kinase domain, highlighting the functional importance of this residue *in vivo*. ENU has a similar mutagenic efficiency to EMS with a less biased spectrum of nucleotide changes^20^,^26^. G:C to T:A transversions, which are common in optogenetic mutagenesis, are rare in ENU as well as EMS. UV/TMP is a less potent mutagen compared to EMS and ENU, and preferentially used to induce insertion and/or deletion mutations^27^. Thus, the unique mutational spectrum of optogenetic mutagenesis complements those of traditional mutagenesis.

Optogenetically induced SNVs have preference towards G:C to T:A or G:C to A:T transversions. It is thought that singlet oxygen mainly reacts with guanine nucleoside as an electrophile and leads to G:C to T:A changes by creating 8-oxo-7,8-dihydro-2'-deoxyguanosine (8-oxodG)^28^. Also, oxidative stress produces oxidized cytosine, 5-hydroxy uracil, which causes G:C to A:T mutations^29^. The spectrum of optogenetically induced mutations is similar to that of singlet oxygen induced mutations using chemicals in bacteria^8^,^28^ and that of spontaneous mutations in wild-type *C. elegans*, which are most likely caused by oxidative DNA damage^18^. These observations support the model that ROS produced by miniSOG is mutagenic to genomic DNA. His-mSOG also induced deletions. ROS including singlet oxygen possibly causes DNA strand breaks^1^,^30^, which can result in deletions and chromosome rearrangements. Indeed, previous studies indicate that histone tagged with the genetically encoded photosensitizer KillerRed can induce double-strand breaks (DSBs) of genomic DNA in cultured cells^31^,^32^,^33^ as assayed by terminal deoxynucleotidyl transferase (TdT)-mediated dUTP Nick-End Labeling (TUNEL) method^34^. There are two major pathways to repair DSBs: error-free homologous recombination (HR) and error-prone non-homologous end joining (NHEJ), which can generate deletions^35^. A recent study also suggests that, instead of NHEJ, polymerase theta-mediated end joining is a major driver of mutations after DSB in *C. elegans* germline^36^. Different mechanisms have been proposed to create different sizes of deletions after DSBs^37^. Therefore, it is possible that deletions caused by His-mSOG mutagenesis ultimately arise from mutagenic DSBs. However, we cannot exclude the possibility that some mutagenic effects of His-mSOG are indirect results of disruption of histone function, as miniSOG-generated ROS can inactivate nearby proteins^7^. In any case, the spectrum of optogenetically induced mutations provides important biological insights into the type of mutations induced by nuclear oxidative stress.

Since miniSOG is genetically encoded, we envisage possible applications of optogenetic mutagenesis in addition to genome-wide random mutagenesis using histone. For example, miniSOG could be fused to a sequence-specific DNA binding protein to specifically mutagenize its unknown targets because potent ROS production from miniSOG appears to be local^3^. Furthermore, optogenetic mutagenesis should be applicable to other organisms such as *Drosophila* and zebrafish.

## Methods

### *C. elegans* maintenance

We maintained *C. elegans* strains on NGM plates at 22.5 °C (ref. 12). Except for routine propagation and crosses using a standard stereomicroscope, we took caution to keep His-mSOG transgenic worms in covered boxes and maintained in standard incubators. Nonetheless, we observed no abnormalities for these strains over a period of one year under repeated exposure to ambient light.

### Plasmids and strains

P*mex-5*-HIS-72::miniSOG-3'UTR(*tbb-2*) was cloned into pCFJ150 backbone for Mos1-mediated single-copy insertion (MosSCI)^38^ using Gibson assembly^39^. The resulting plasmid pCZ886 contains 488 bp of 5' region of *mex-5* gene plus *his-72* coding region with a SRPVAT linker inserted between *his-72* and miniSOG, and 330 bp of 3' region of *tbb-2* gene. pCZ886 plasmid was injected at 50 ng μl^−1^ into EG8080 *oxTi444 [ttTi5605 + NeoR(+) + unc-18(+)] unc-119(ed3)* to generate a single-copy-inserted transgene *juSi164[Pmex-5-HIS-72::miniSOG-3'UTR(tbb-2), Cb-unc-119(+)]* III using a standard Universal MosSCI insertion method^38^. We detected no visible fluorescence in the germline under the epifluorescence microscope with 488-nm light, generally consistent with the weak fluorescence of miniSOG. CZ20310 *juSi164 unc-119(ed3) III* strain was used for optogenetic mutagenesis to isolate mutants with visible phenotypes. CZ20638 *juSi164 unc-119(ed3)III; juIs1[Punc-25-SNB-1::GFP]IV; rpm-1(ju44)V; syd-2(ju37)X* was generated by crossing CZ20310 to CZ1338 *juIs1; ju44; ju37* and used for the mutagenesis in the suppressor screen of *rpm-1(lf); syd-2(lf).* The *rol-6(su1006)* plasmid (pRF4)^24^ was injected into CZ20310 at 50 ng/μl to generate CZ22318 *juSi164 unc-119(ed3); juEx6771[rol-6(su1006)]. unc-119(ed3)* was rescued by Cb-*unc-119(+)* on the pCZ886 plasmid in all the *juSi164*-carrying strains. Supplementary Table 1 lists the strains used in this study.

### Blue light illumination setup

We used a blue Ultra High Power LED (UHP-LED-460) light source (460±5 nm, Prizmatix, Southfield, MI) connected to a digital function generator/amplifier (PI-9587C, PASCO, Roseville, CA) with a TTL cable. The light source was fixed approximately 10 cm above the plate using a custom-made stage. The sine wave of light was illuminated at 4 Hz with 65% of the maximum power (>2.3 W). To prepare animals for optogenetic mutagenesis, a filter paper with a 25 × 25 mm hole was soaked with 100 mM CuCl_2_ and placed onto a 60 mm NGM plate without bacteria to restrict animals within the illuminated area. Dozens of L4, non-gravid (3–7 h post-L4), or gravid (8–12 h post-L4) young adult worms as P_0_ were picked and transferred to new plates. The blue light intensity on the plate was measured by a photometer to be 2.0 mW/mm^2^ under continuous illumination.

### Mutagenesis and mutation frequency analysis

In our standard protocol, we illuminated gravid young adult animals with blue light for 30 min at 4 Hz. As a control we put the worms on the CuCl_2_ plates that were wrapped with foil without exposure to blue light. After light treatment single P_0_ worms were transferred to a new plate daily to count the number of F_1_ progeny. For lifespan measurement, 20 light-treated worms were transferred to a new plate every two days and the number of surviving worms was plotted as a function of days after egg-laying. For the screen in the wild-type background, CZ20310 *juSi164 unc-119(ed3)* worms were treated with blue light and F_2_ worms with visible mutant phenotypes were isolated. Phenotypes were scored as heritable mutants if they were propagated to the F_3_ or subsequent generations. For the suppressor screen of *rpm-1(lf); syd-2(lf)*, CZ20638 *juSi164 unc-119(ed3); juIs1; rpm-1(lf); syd-2(lf)* or CZ1338 *juIs1; rpm-1(lf); syd-2(lf)* worms were treated with blue light or 50 mM EMS (Sigma) following a standard protocol^12^, respectively. We recovered 20 worms after blue light or EMS treatment and transferred individual worms on single seeded plates. We scored the number of plates carrying at least a few well moving F_2_ worms. From the number of the plates carrying suppressors and the average brood size in each condition, the frequency of the mutations was calculated as:


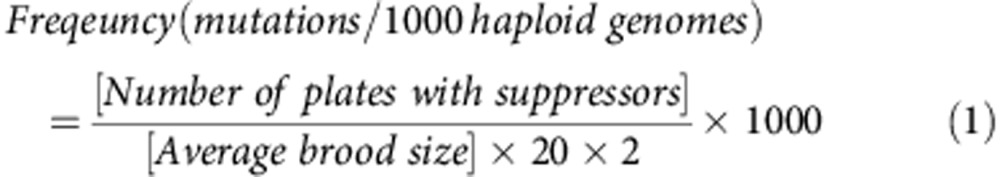


The average mutation rate using 50 mM EMS is known to be about one loss-of-function mutation per gene in 2,000 haploid genomes^12^. Since there are six major loci for the *rpm-1(lf); syd-2(lf)* suppressor screen, the predicted frequency is 3.0 suppressor mutations/1,000 haploid genomes. Our average mutation frequency (3.09±0.52 /1,000 haploid genomes, *n*=4 experiments, total ∼21,000 mutagenized haploid genomes) matched the estimated value, validating our method to calculate the frequency of mutations (Fig. 2d). It should be noted that equation (1) assumes all mutants from the same P_0_ are siblings because the chance to get more than one mutants should be reasonably low.

### Genetic mapping

The Dumpy mutation *ju1157* was linked to *juSi164* (III: -0.85) and *unc-119* (*ed3*, III: +5.59) during outcrossing (Fig. 4a). The Uncoordinated mutation *ju1158* was mapped to the right arm of the chromosome I by SNV mapping using Hawaiian CB4856 strain (Fig. 4a). The molecular lesions of *ju1157* and *ju1158* were found by WGS and confirmed by Sanger sequencing. The suppressors of *rpm-1(lf); syd-2(lf)* were subjected to complementation tests with known alleles for the identification of loci^14^. The molecular lesions were then determined by PCR and Sanger sequencing.

### Whole-genome sequencing

CZ20638 was treated with and without light using the standard protocol. Two non-suppressed worms and three suppressed worms were isolated from F_2_ generations of independent untreated and light-treated plates, respectively. To drive existing mutations to homozygosity single hermaphrodite from these strains and the parental CZ20638 strain were propagated by self-fertilization for five generations. The number of changes described below is likely an underestimate because some mutations may be lost in the singling process. Genomic DNA was prepared from three 15-cm plates each using Puregene Cell and Tissue Kit (Qiagen, Valencia, CA) according to the manufacturer's instruction. Purified genomic DNA was sequenced using the 90-bp paired-end Illumina Hiseq 2,000 at Beijing Genomics Institute (BGI Americas) with 20-fold coverage for 95% of nucleotides. The obtained raw sequences were mapped to the *C. elegans* reference genome (Wormbase release 220) using BWA^40^ in the Galaxy platform^41^. The threshold of the ‘quality score' in Galaxy for reliable SNVs was set to 200 as previously suggested^17^. GATK tools^42^ were used for SNV and small indel calling. By subtracting the variants in the original strains and common variants among the strains in our laboratory, we determined unique homozygous SNV variants for strains with and without light exposure. Unique uncovered regions were defined as those not overlapping with uncovered regions in other strains for which we had WGS data. Unique uncovered regions of > 200-bp were analyzed using Microsoft Excel and confirmed by PCR and Sanger sequencing.

### Chromosomal integration

We used CZ22318 *juSi164 unc-119(ed3); juEx6771[rol-6(su1006)]* because of its low transgene transmission rate (∼20 %) and used it for chromosomal integration by optogenetic mutagenesis. CZ22318 worms were treated with the 30-min blue light protocol at 4 Hz. Two hundred F_1_ roller worms were singled on the seeded plates (F_1_ plates). The F_2_ worms were singled from the F_1_ plates with >75% roller worms. Some F_2_ animals showed 100% transmission of the transgene in the following generation, suggesting that the transgene may have been integrated chromosomally. To distinguish chromosomal integrations from transgene stabilization, possible integrations were outcrossed to CZ8835 *muIs32[Pmec-7-GFP]* males to examine segregation in the F_2_ generation.

### Statistical analysis

Two-tailed *t*-test and one-way ANOVA with Bonferroni's multiple comparison were used for statistical analyses of one pair and more than one pair of comparisons, respectively, using GraphPad Prism 5.0 software (GraphPad Software, La Jolla, CA).

## Additional information

**Accession codes:** The whole genome sequencing reads from the mutageneised *C. elegans* have been submitted to the NCBI Sequence Read Archive with the accession codes CZ20638 SRS1107346, CZ20638 NC1 SRS1103686, CZ20638 NC2 SRS1107318, CZ22336 SRS1107342, CZ22337 SRS1107340 and CZ22338 SRS1107339.

**How to cite this article:** Noma, K & Jin, Y. Optogenetic mutagenesis in *Caenorhabditis elegans*. *Nat. Commun.* 6:8868 doi: 10.1038/ncomms9868 (2015).

## Supplementary Material

Supplementary Information Supplementary Figures 1-2 and Supplementary Table 1

## Figures and Tables

**Table 1:** Optogenetically induced homozygous non-synonymous SNVs.

**Chr**	**Position (base)**	**Wild type**	**Mutant**	**Gene**	**WT/Mut AA**
CZ22336 *dlk-1(ju1290); juSi164 unc-119(ed3); juIs1; rpm-1(lf); syd-2(lf)*					
I	4725957	G	T	*ugt-27*	G/C
I	7011255	G	C	*H06O01.2*	D/E
I	7219828	A	G	*nhr-23*	Y/C
I	12570028	C	A	*dlk-1*	V/F
I	12650161	G	T	*H28O16.2*	R/S
I	13657411	G	T	*W09C5.7*	G/V
X	16073314	G	T	*col-187*	R/I
X	16927569	C	A	*C30E1.2*	N/K
					
CZ22337 dlk-1(ju1291)* *juSi164 unc-119(ed3); juIs1; rpm-1(lf); syd-2(lf)*					
I	2415913	G	T	*F23C8.13*	G/W
I	10465587	C	G	*duo-3*	C/S
IV	8287065	C	T	*K07H8.10*	A/T
IV	8656791	T	C	*C49C8.1*	X/R
IV	11587778	G	T	*F12F6.1*	K/N
V	16796857	G	T	*W06H3.3*	Q/H
X	9552913	C	A	*F49E2.5*	A/D
X	11760549	C	A	*lact-7*	*P*/T
X	14091587	C	A	*dsl-4*	P/H
					
CZ22338 *juSi164 unc-119(ed3); pmk-3(ju1292) juIs1; rpm-1(lf); syd-2(lf)*					
I	9421262	G	T	*pabp-2*	R/S
II	2791746	G	A	*F08D12.1*	V/I
III	132562	G	A	*C29F9.1*	G/R
III	1754908	A	C	*Y46E12A.3*	L/W
IV	8141724	G	T	*pmk-3*	G/V
V	4750824	C	A	*C18G1.6*	H/N
V	10920642	A	G	*nas-31*	L/P

^*^ju1291 is likely a chromosome deletion and/or rearrangement in *dlk-1*.

**Table 2:** Optogenetically induced *rpm-1(lf); syd-2(lf)* suppressors.

**Allele**	Gene*	**NT change (unspliced)**	**NT change (spliced)**	**AA change**
*ju1202*	*dlk-1*	G2552T	G788T	C263F
*ju1209*	*dlk-1*	G3665T	G1570T	G524X
*ju1211*	*dlk-1*	G2442T	G678T	W226C
*ju1290*†	*dlk-1*	G2764T	G904T	V302F
*ju1203*	*dlk-1*	2314-Tc3-2315		
*ju1210*	*dlk-1*	44-59 deletion		
*ju1218*	*dlk-1*	ND‡		
*ju1220*	*dlk-1*	ND‡		
*ju1291*†	*dlk-1*	ND‡		
*ju1217*	*dlk-1*	ND§		
*ju1207*	*pmk-3*	C2364A	C506A	T169K
*ju1213*	*pmk-3*	G2010C	G450C	K150N
*ju1215*	*pmk-3*	G3508C	G1351C	A451P
*ju1292*†	*pmk-3*	G2520T	G617T	G206V
*ju1208*	*pmk-3*	1180-1499 deletion		
*ju1222*	*pmk-3*	1248 deletion		
*ju1212*	*mkk-4*	G135T		
*ju1221*	*mkk-4*	C736A	C521A	T174K
*ju1216*	*mak-2*	C3276G	C809G	T270R
*ju1219*	*mak-2*	1177-1274 deletion		
*ju1214*	*cebp-1*	C719A	C528A	Y176X

^*^Causative genes were determined by complementation tests.

^†^The strains were analyzed by WGS.

^‡^Some PCR products were not obtained when amplified from *dlk-1* locus using multiple pairs of primers, suggesting that they have a deletion and/or chromosomal rearrangement.

^§^No SNVs were found in the entire coding sequences of *dlk-1*, suggesting a mutation in regulatory regions.
