# Supplementary material for: Optogenetic mutagenesis in Caenorhabditis elegans
Source: Nat Commun. 2015 Dec 3;6:8868. doi: 10.1038/ncomms9868 (PMC4686824; doi:10.1038/ncomms9868)
Supplement: Supplementary Information — Supplementary Figures 1-2 and Supplementary Table 1 [file ncomms9868-s1.pdf]

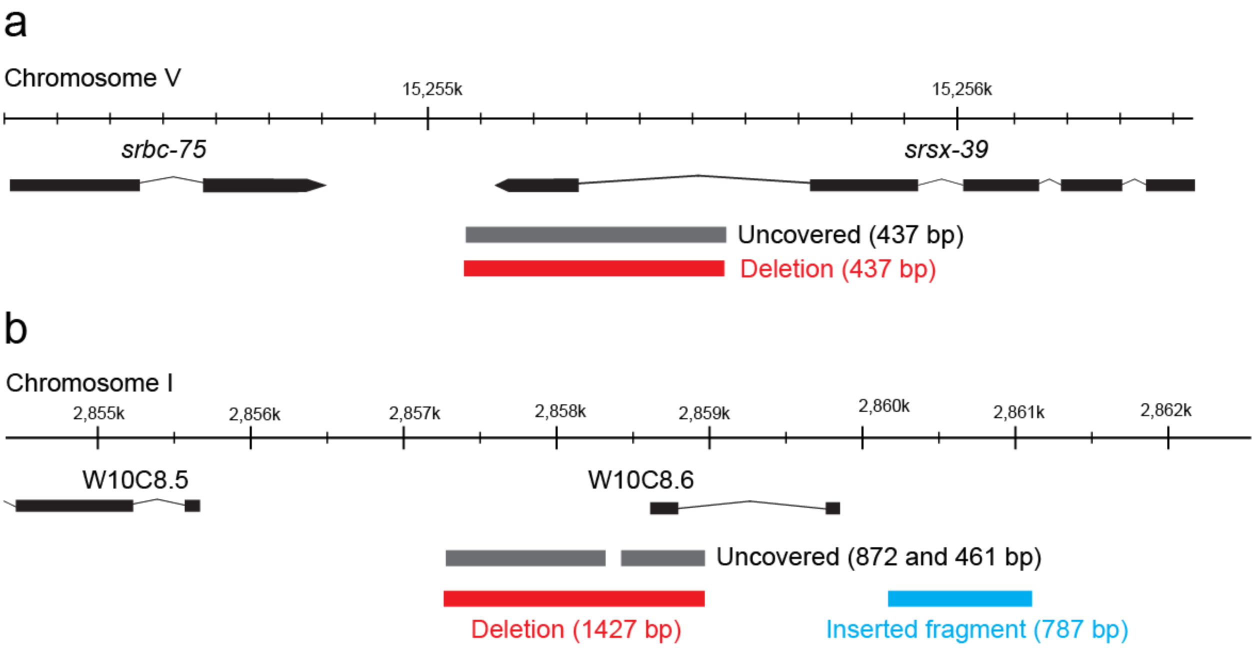

Supplementary Fig. 1 Verification of optogenetically induced deletions

Genomic locations of uncovered regions revealed by WGS analysis of optogenetically induced *rpm-1(lf); syd-2(lf)* suppressors. Molecular lesions were confirmed by PCR and Sanger sequencing. (a) A 437-bp uncovered region (gray bar) in CZ22336 *dlk-1(ju1290); juSi163 ed3; juIs1; rpm-1(lf); syd-2(lf),* which was confirmed by PCR and Sanger sequencing to be a 437-bp deletion (red bar). (b) Two adjacent uncovered regions (gray bars) in CZ22334 *juSi163 ed3; pmk-3(ju1291); juIs1; rpm-1(lf); syd-2(lf)*, which was confirmed by PCR and Sanger sequencing to be a 1427-bp deletion (red bar) with a 787-bp insertion from neighboring region to the deleted region (light blue bar).

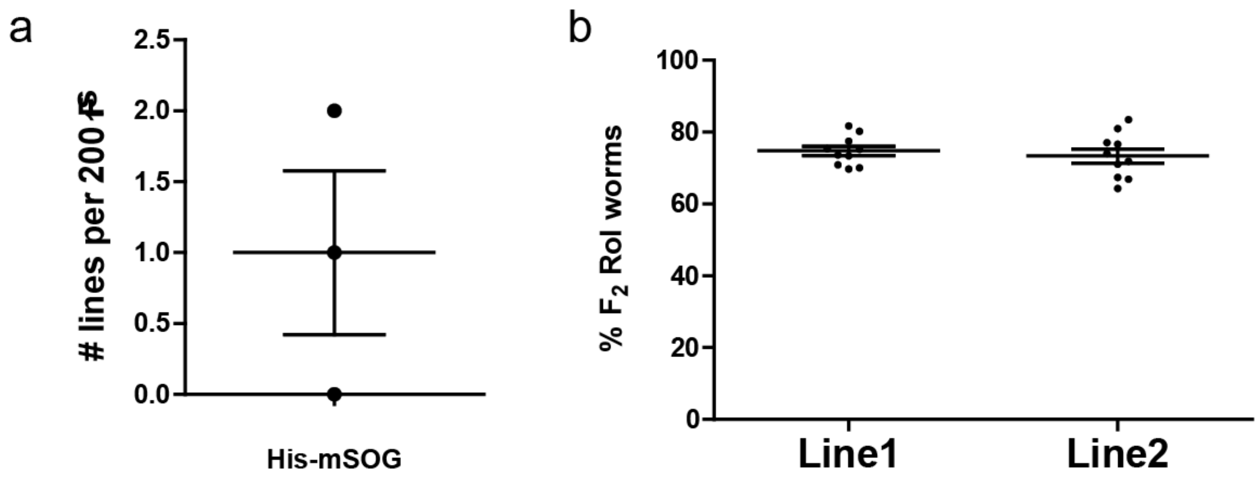

Supplementary Fig. 2 His-mSOG mutagenesis can induce integration of multi-copy transgenes

Parental worms expressing His-mSOG in the germline as well as multi-copy transgenes are treated with blue light. (a) Number of lines obtained from 200 F_1 worms. n= 3 independent experiments. (b) Ratio of roller worms among F_2 worms. Since *rol-6(su1006)* is a dominant roller phenotype, 75% of roller F_2 worms indicate a Mendelian segregation. Ten F_1 worms were examined for two independently isolated lines. Error bars indicate S.E.M. for the both panels.

Supplementary Table 1 Strain list

Strain	Genotype
CZ20310	*juSi164[Pmex-5-HIS-72::miniSOG::3'UTR(tbb-2) + Cb-unc-119(+)*] unc-119(ed3)III*
CZ20372	*dpy-1(ju1157) juSi164 unc-119(ed3)III*
CZ20373	*unc-122(ju1158)I; juSi164 unc-119(ed3)III*
CZ1338	*juIs1[Punc-25-SNB-1::GFP]IV; rpm-1(ju44)V; syd-2(ju37)X*
CZ20638	*juSi164 unc-119(ed3)III; juIs1IV; rpm-1(ju44)V; syd-2(ju37)X*
CZ20843	*dlk-1(ju1202)I; juSi164 unc-119(ed3)III; juIs1IV; rpm-1(ju44)V; syd-2(ju37)X*
CZ20844	*dlk-1(ju1203)I; juSi164 unc-119(ed3)III; juIs1IV; rpm-1(ju44)V; syd-2(ju37)X*
CZ21092	*juSi164 unc-119(ed3)III; pmk-3(ju1207) juIs1IV ;rpm-1(ju44)V; syd-2(ju37)X*
CZ21093	*juSi164 unc-119(ed3)III; pmk-3(ju1208) juIs1IV; rpm-1(ju44)V; syd-2(ju37)X*
CZ21094	*dlk-1(ju1209)I; juSi164 unc-119(ed3)III; juIs1IV; rpm-1(ju44)V; syd-2(ju37)X*
CZ21095	*dlk-1(ju1210)I; juSi164 unc-119(ed3)III; juIs1IV; rpm-1(ju44)V; syd-2(ju37)X*
CZ21096	*dlk-1(ju1211)I; juSi164 unc-119(ed3)III; juIs1IV; rpm-1(ju44)V; syd-2(ju37)X*
CZ21097	*juSi164 unc-119(ed3)III; juIs1IV; rpm-1(ju44)V; mkk-4(ju1212) syd-2(ju37)X*
CZ21098	*juSi164 unc-119(ed3)III; pmk-3(ju1213) juIs1IV; rpm-1(ju44)V; syd-2(ju37)X*
CZ21099	*juSi164 unc-119(ed3)III; juIs1IV; rpm-1(ju44)V; cebp-1(ju1214) syd-2(ju37)X*
CZ21100	*juSi164 unc-119(ed3)III; pmk-3(ju1215) juIs1IV; rpm-1(ju44)V; syd-2(ju37)X*
CZ21101	*juSi164 unc-119(ed3)III; mak-2(ju1216) juIs1IV; rpm-1(ju44)V; syd-2(ju37)X*
CZ21102	*dlk-1(ju1217)I; juSi164 unc-119(ed3)III; juIs1IV; rpm-1(ju44)V; syd-2(ju37)X*
CZ21103	*dlk-1(ju1218)I; juSi164 unc-119(ed3)III; juIs1IV; rpm-1(ju44)V; syd-2(ju37)X*
CZ21104	*juSi164 unc-119(ed3)III; mak-2(ju1219) juIs1IV; rpm-1(ju44)V; syd-2(ju37)X*
CZ21105	*dlk-1(ju1220)I; juSi164 unc-119(ed3)III; juIs1IV; rpm-1(ju44)V; syd-2(ju37)X*
CZ21106	*juSi164 unc-119(ed3)III; juIs1IV; rpm-1(ju44)V;mkk-4(ju1221) syd-2(ju37)X*
CZ21107	*juSi164 unc-119(ed3)III; pmk-3(ju1222) juIs1IV; rpm-1(ju44)V; syd-2(ju37)X*
CZ22336	*dlk-1(ju1290)I; juSi164 unc-119(ed3)III; juIs1IV; rpm-1(ju44)V; syd-2(ju37)X*
CZ22337	*dlk-1(ju1291)I; juSi164 unc-119(ed3)III; juIs1IV; rpm-1(ju44)V; syd-2(ju37)X*
CZ22338	*juSi164 unc-119(ed3)III; pmk-3(ju1292) juIs1IV; rpm-1(ju44)V; syd-2(ju37)X*
CZ22318	*juSi164 unc-119(ed3)III; juEx6771[rol-6(su1006)]*

**juSi164* also contains Neo(+) and *unc-18*(+) derived from MosSCI insertion.
